# *Mycobacteria avium*-related peritonitis in a patient undergoing peritoneal dialysis: case report and review of the literature

**DOI:** 10.1186/s12882-021-02544-2

**Published:** 2021-10-19

**Authors:** Jifang Lu, Zhou Jiang, Ling Wang, Shan Mou, Hao Yan

**Affiliations:** 1 Department of Nephrology, Ningbo Hangzhou Bay Hospital, Ningbo, Zhejiang China; 2 Department of Pathology, Ren Ji Hospital, Shanghai Jiao Tong University School of Medicine, Shanghai, China; 3 Department of Nephrology, Ren Ji Hospital, Shanghai Jiao Tong University School of Medicine, No.160, Pujian Road, Pudong District, Shanghai, 200127 P.R. China

**Keywords:** Peritoneal dialysis-related peritonitis case report, Nontuberculous mycobacteria, *Mycobacteria avium*, Chylous ascites, Adenosine deaminase

## Abstract

**Background:**

*Mycobacteria avium* (*M. avium*) is a species of ubiquitous slowly growing nontuberculous mycobacteria. It causes opportunistic infections. However, *M. avium*-related peritonitis in peritoneal dialysis (PD) patients is rare.

**Case presentation:**

A 51-year-old female end-stage kidney disease patient undergoing PD was admitted for a noninfectious complication. She presented catheter exit site drainage and slightly increased PD effluent white cell count (WCC) with polymorphonuclear predominance on admission. Exit site infection and PD-related peritonitis were diagnosed. Repeated cultures of effluent and drainage were negative. Initial empirical antibiotics and further adjustment were not rewarding. PD was terminated 2 weeks later, however, shortly the patient developed stupor, high fever, peritoneal irritation, and spontaneous chylous ascites, and showed elevated ascitic adenosine deaminase (ADA). The manifestations persisted and the patient’s general condition deteriorated despite intensified antibiotic therapy. Massive parallel sequencing identified *M. avium* in ascites on hospital day 25, and 4-drug treatment with azithromycin, amikacin, rifampin, and ethambutol was initiated. Nevertheless, the patient died from sepsis on hospital day 30.

**Conclusions:**

We report a case of PD-related *M. avium* peritonitis. Prolonged culture-negative peritonitis, chylous ascites, and elevated ascitic ADA may hint the possibility of mycobacterial infections. Diagnostic method allowing prompt identification of the pathogen is warranted. The prognosis can be extremely poor, and the prophylaxis and treatment should be better defined.

## Background

*Mycobacteria avium* (*M. avium*) is a species of slowly growing nontuberculous mycobacteria (NTM). *M. avium*, *M intracellulare*, and an increasing number of newly identified species comprise *M. avium* complex (MAC). Most laboratories are unable to differentiate the exact species because of lack of molecular methods required. MAC is widespread in the environment, and can be an opportunistic pathogen [1].

Immunodeficient patients are predisposed to MAC infections, particularly those with acquired immune deficiency syndrome (AIDS) [2]. Lung disease is the most common form of MAC infections, accounting for 80% of NTM-related pulmonary disease. MAC can also invade the lymph nodes, bones, joints, skin, and soft tissue to cause extrapulmonary infections, and can spread systemically, resulting into disseminated infections [1].

End stage kidney disease (ESKD) patients are usually affected by suppressed immunity, and infections by opportunistic pathogens are more frequent than in general populations [3]. However, reports on peritoneal dialysis (PD)-related MAC infections are sparse. Herein, we describe a case of *M. avium* peritonitis in a patient undergoing PD. The literature is reviewed regarding the presentation, diagnosis, treatment, and outcomes of this condition.

## Case presentation

A 51-year-old lady visited our center in August 2020 for the first time. She was congenitally deaf and mute, and commenced continuous ambulatory PD in 2015 at another facility without knowing the etiology of ESKD. During the 5 years on PD, the patient had not been regularly followed up. She was anuric, with manifestations of fatigue, malaise, anorexia, itchiness, and restless legs. There was no sign of infections. Laboratory exams showed significant anemia, hypoalbuminemia, hypokalemia, and weekly Kt/V_urea_ of 1.55. The symptoms were improved after increasing the PD dose and medications adjustment. However, 1 month later she was admitted due to uncontrolled hypertension as a result of incompliance with anti-hypertensive therapy.

On admission, the patient was afebrile, conscious but weak, with no obvious abdominal pain. Physical examination revealed no significance except a few collections at the PD catheter exit site. There was no visible turbidity in PD effluent despite an unexpected effluent white cell count (WCC) of 277/μL with 97% polymorphonuclear. CRP (28.83 mg/L) and procalcitonin (1.190 ng/mL) were slightly elevated; peripheral WCC, lipopolysaccharide, and (1 → 3)-β-D glucan were normal; tests for human immunodeficiency virus and *M. tuberculosis* antibody were negative; no organism was noted on Gram stain for PD effluent or exit site drainage; sonography and CT scan showed no signs of PD catheter tunnel infection, pneumonia, endocarditis, cirrhosis, or other pathology of celiac viscera. PD-related peritonitis and concomitant exit site infection were suspected. Intensified exit site care and empirical treatment with intraperitoneal vancomycin, amikacin and oral fluconazole were initiated immediately after culture samples were sent on hospital day 1.

No bacterium or fungus was isolated from repeated cultures of PD effluent and exit site drainage, and the case was treated as culture-negative peritonitis. The effluent WCC was persistently around 200/μL with neutrophil predominance during the first week, and there was no improvement after replacement with intraperitoneal cefepime and oral rifampin during the second week. Although it was strongly recommended, the patient adamantly refused catheter removal.

On hospital day 14 PD was terminated since the effluent WCC increased to 515/μL. A central venous catheter was inserted uneventfully on hospital day 15, then hemodialysis was performed every other day. Surprisingly, she became stuporous on hospital day 16. Blood tests showed no significant disorder of homeostasis, neither CT scan found any evidence of acute cerebrovascular disease or intracranial mass. Meningitis was suspected. Unfortunately, the family members refused invasive procedures including lumbar puncture and PD catheter removal.

**Fig. 1:**
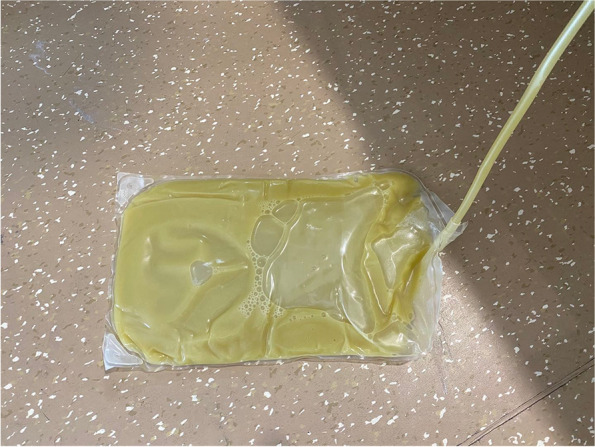
Yellow chylous ascites drained via the peritoneal dialysis (PD) catheter after PD cessation

On hospital day 17, the patient had a high fever and signs of peritoneal irritation. Yellow chylous ascites (Fig. 1) was drained through the PD catheter after detection of intraperitoneal collections by CT scan. Exams for ascites showed WCC of 2700/μL with 65% neutrophil and 35% lymphocyte, as well as elevated triglyceride (3.1 mmol/L) and adenosine deaminase (ADA, 94 IU/L), but normal amylase and negative acid-fast bacilli (AFB) staining. Blood CRP ascended to 219 mg/L, while blood and ascites cultures remained negative. Despite use of antibiotics including cefoperazone, levofloxacin, linezolid, and meropenem, the fever and chylous drainage persisted.

**Fig. 2:**
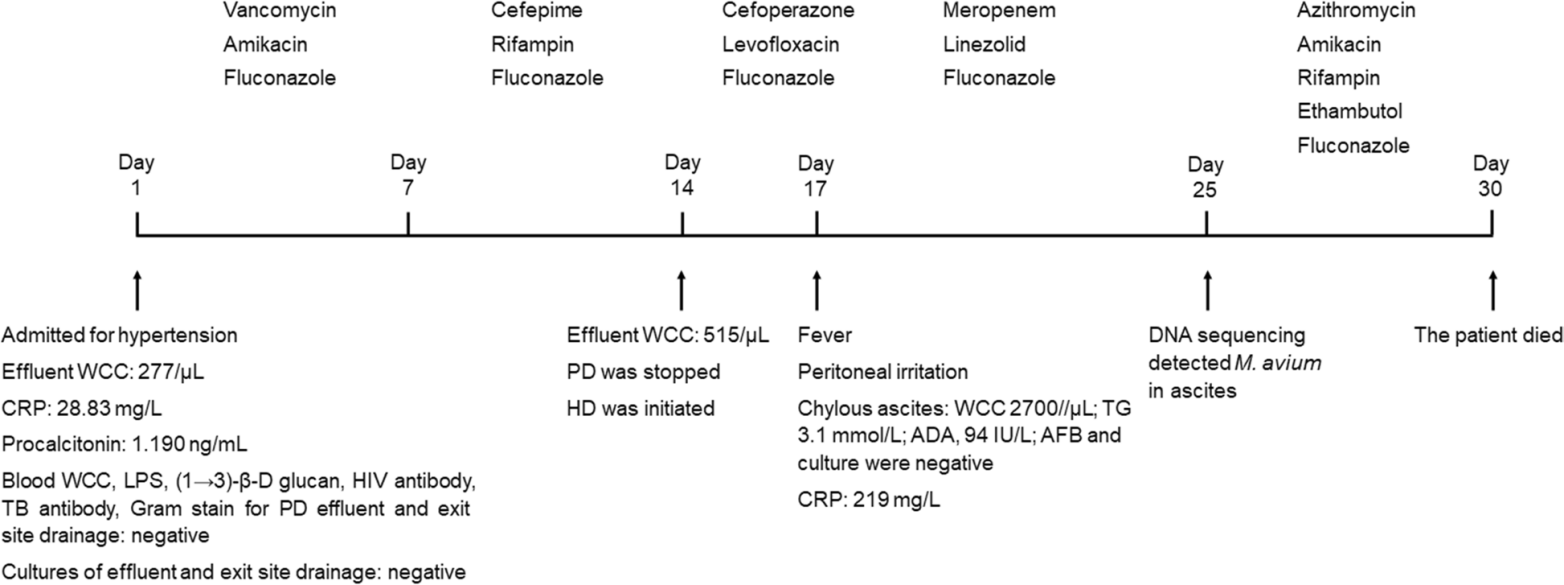
Summary of the hospitalization course. WCC: white cell count; LPS: lipopolysaccharide; HIV: human immunodeficiency virus; TB: tuberculosis; PD: peritoneal dialysis; HD: hemodialysis; TG: Triglyceride; ADA: adenosine deaminase; AFB: acid-fast bacilli

On hospital day 23, an ascites sample was sent for massive parallel sequencing. Two days later *M. avium* was reported, and intravenous azithromycin (500 mg per day) and amikacin (100 mg per day), together with oral rifampin (600 mg per day) and ethambutol (750 mg thrice a week) were prescribed. Within the following days, abdominal tenderness subsided and drainage was apparently reduced, but the hyperpyrexia sustained and the general conditions continued to deteriorate. On hospital day 30, the patient expired from sepsis. The course of hospitalization is summarized in Fig. 2.

## Discussion and conclusion

Data on PD-related NTM peritonitis is increasing, however, majority of the cases are caused by rapidly growing mycobacteria such as *M. fortuitum* and *M. abscessus*, etc. [4, 5], while MAC and other slowly growing NTM-related peritonitis in PD population has rarely been reported. Song et al. reviewed 57 episodes of PD-associated NTM peritonitis, and found that MAC was responsible for only 6 episodes [6]. To the best of our knowledge, previous publications reported 10 PD patients diagnosed with MAC peritonitis [7–15].

The characteristics of the patients including the one we present are summarized in Table 1. There were 3 patients with a history of transplantation, 2 with systemic lupus erythematosus, and 1 with AIDS. Although the present patient did not have such comorbidities, she was obviously underdialyzed, malnourished, and probably with immune deficiency. Exact causes of the infection were commonly untraceable, except in a pediatric patient contamination was identified [15]. Fever was observed in at least 8 patients, and it was usually over 38.5 °C. Coincident PD exit site infection was reported in 4 patients. In majority of the cases, the PD effluent WCC at peritonitis onset was slightly elevated. At least half of the patients showed a polymorphonuclear predominance, which is in accordance with the manifestation of other PD-related NTM peritonitis [4] and may distinguish from tuberculous peritonitis [16]. AFB smear was positive in 6 patients. Granulomas or caseous lesions in peritoneal cavity were noticed by imaging or pathological examinations in multiple cases. Eight cases were identified by cultures, while the rest including the present one were diagnosed according to the results of DNA sequencing. In 8 cases with available data, the time spans between peritonitis onset and identification of MAC infection ranged from 6 days to 8 weeks, with a mean of 28 days.

**Table 1:** Reported cases of PD-related MAC peritonitis

References	Sex	Age (years)	Primary disease or comorbidity	Fever	ESI	Initial PDF WCC and differential	PDF AFB smear	Imaging/pathological findings	diagnostic method	Medications	Outcome
Pulliam et al. [7]	M	16	KT	+	+	1900/μL PMN 80%	+	Pathological study: granulomas in peritoneal cavity, lung, and liver	PDF culture	Isoniazid Rifampin Amikacin	HD and CVD-related death
Perlino [8]	F	49	HTN and DCM	NR	+	NR	+	Laparotomy: abdominal caseous material and thickened peritoneum	PDF culture	Ciprofloxacin Ethambutol Rifampin Amikacin	HD and CVD-related death
Perlino [8]	F	41	LN and KT	NR	+	NR	+	Laparotomy: peritoneum with tuberculosis-like lesions	PDF culture	Ciprofloxacin Ethambutol Rifampin Amikacin	HD and Infection-related death
Perazella et al. [9]	M	45	HIV nephropathy	+	–	161/μL PMN predominance	+	CT: abdominal wall abscess unrelated to the PD catheter	PDF culture	Clarithromycin Clofazimine	Infection-related death
Irizarry et al. [10]	M	54	FSGS	+	–	90/μL Lymphocyte 77% Mononuclear 33%	+	Laparotomy: multiple areas of purulence throughout the peritoneum	PDF culture	Amikacin and 3 unknown antibiotics	HD
Ferrara et al. [11]	F	16	LN	+	–	226/μL PMN 18% Lymphocyte 33% Macrophage 41%	–	Sonography: periportal edema, hepatosplenomegaly, possible granulomas within the spleen	PDF culture	Azithromycin Ethambutol Rifabutin	HD
Falcone et al. [12]	M	64	DKD	–	–	160/μL PMN predominance	NR	CT: inflammatory changes in the omentum	PDF culture	Clarithromycin Ethambutol Rifabutin	HD and Infection-related death
Chung JW et al. [13]	F	69	Left renal agenesis and HTN	+	–	170/μL PMN 88%	–	CT: Multiple lymphadenopathies and multiple masses in the splenic region	Bone marrow DNA sequencing	Isoniazid Rifampin Pyrazinamide Ethambutol	HD and CVD-related death
Miyashita et al. [14]	F	11	HSCT	+	–	613/μL Mononuclear 82% PMN 18%	+	No specific finding on CT	PDF DNA sequencing	Clarithromycin Ethambutol Levofloxacin	HD
Yakota et al. [15]	M	4	Hypoplastic kidneys	+	–	775/μL PMN 74%	NR	No specific finding on CT	PDF culture	Clarithromycin Ethambutol Rifampin	HD and KT
The present case	F	51	Underdialyzed	+	+	277/μL PMN 82% Mononuclear 18%	–	No specific finding on CT or sonography	PDF DNA sequencing	Azithromycin Ethambutol Rifampin Amikacin	HD and Infection-related death

*AFB* Acid-fast bacilli, *CT* Computed Tomography, *CVD* Cardiovascular disease, *DCM* Dilated cardiomyopathy, *DKD* Diabetic kidney disease, *ESI* Exit site infection, *FSGS* Sclerosing glomerulonephritis, *HD* Hemodialysis, *HIV* Human immunodeficiency virus, *HSCT* Hematopoietic stem cell transplantation, *HTN* Hypertension, *KT* Kidney transplantation, *LN* Lupus nephritis, *NR* Not reported, *PD* Peritoneal dialysis, *PDF* Peritoneal dialysis fluid, *PMN* Polymorphonuclear

In contrast with the previously reported MAC peritonitis in PD patients, our case is characterized by chylous ascites. Steinemann et al. reported that in overall cases of chylous ascites, 10% were attributed to mycobacterial infections, both tuberculous mycobacteria and NTM were involved [17]. A systemic review by Baldolli et al. showed that chylous ascites was observed in 56% of MAC peritonitis patients with various comorbidities, especially those with AIDS [18]. Other common causes of chyloperitoneum among PD patients including malignance, trauma, cirrhosis, use of calcium channel blockers, and pancreatitis etc. were excluded in the present case. The mechanism remains obscure, which may implicate obstruction of the lymphatic system due to enlarged lymph nodes or lymph nodal fibrosis by mycobacterial infection [19, 20].

The other novel finding in the present case is the elevated ascitic ADA level. Production of ADA in bodily fluids reflects the presence of activated T lymphocytes and monocytes. As demonstrated by two meta-analyses, ADA in ascites is sensitive and specific to diagnose tuberculous peritonitis [21, 22], and it is supported by a case report on PD-related tuberculous peritonitis [23]. However, lung diseases due to NTM including MAC infection also present increased ADA in pleural effusion [24]. Although the validity has not been established [25, 26], our finding suggests the potential role of ADA to indicate mycobacterial peritonitis among PD patients. One problem is that a negative result is possible due to dilution when dwelled PD fluid exists.

The actual incidence of MAC peritonitis may be underestimated as a result of difficulties in diagnosis [1]. Although AFB staining is easy to perform, it has low sensitivity and cannot distinguish MAC from other mycobacteria. The identification of MAC conventionally relies on cultures in solid and liquid medium. However, it is time consuming, usually takes several weeks, and negative culture is not uncommon. Molecular methods including gene sequencing can accelerate the process of identification, while the accessibility and affordability remain critical.

There have been no large trials to guide the treatment of extrapulmonary MAC infections including MAC peritonitis. The latest International Society for Peritoneal Dialysis guideline on peritonitis suggests individualized protocols for NTM peritonitis based on susceptibility testing and timely PD catheter removal in the setting of suboptimal treatment response, nevertheless, no specific medications are suggested [27]. In addition, the pathogens are not covered by most of the recommended empirical antibiotics for PD-related peritonitis. The experience of treatment for PD-related MAC peritonitis is limited, and relevant antibiotic regimens may be extrapolated from recommendations for other MAC infections. The statement by the American Thoracic Society and the Infectious Diseases Society of America published in 2007 recommends a combination of a macrolide (clarithromycin or azithromycin) and ethambutol, with or without a rifamycin (rifabutin or rifampin) to treat disseminated infections [28]. Recently, a panel of international respiratory medicine and infectious diseases societies established a guideline on NTM pulmonary disease [29]. It advocates a 3-drug or 4-drug regimen for severe or refractory MAC lung disease, and the preferred drugs include azithromycin, ethambutol, rifampin, and intravenous amikacin or streptomycin. The optimal antibiotic course remains unaddressed, and it is suggested to be at least 12 months after culture conversion.

Baldolli et al. showed that the mortality in overall MAC peritonitis was as high as 50% [18]. In PD population, although more than 85% of the NTM peritonitis patients could survive [6], 7 out of 11 MAC peritonitis patients died according to our review. Death directly attributed to MAC infection usually occurred within 3 months after peritonitis onset, and cardiovascular event was another important cause of death in the context of MAC peritonitis. All the survivors had to remove the PD catheter, switch to hemodialysis, and received anti-mycobacterial therapy for 12 or 18 months. One patient successfully underwent kidney transplant 8 months after completion of anti-mycobacterial treatment [15].

We speculate that PD catheter remaining in situ contributed to the clinical deterioration and eventual death in the patient. Timely catheter removal is essential to treat refractory peritonitis, and it is usually required for NTM peritonitis [27]. The formation of biofilms is a common pathogenic factor of mycobacteria, and it may play an important role in antimicrobial resistance [30]. For this case, it was possible that after PD cessation biofilms on the catheter provoked the intraabdominal infection and inflammation. Excessive production and retention of inflammatory factors in peritoneal cavity might cause the worsened abdominal symptoms and fever. Another unanswered question was the change of the patient’s conscious state. Whether it was a consequence of disseminated *M. avium* infection involving the central nervous system was unclear.

In conclusion, *M. avium* or MAC-related peritonitis in patients undergoing PD is rare. Culture-negative peritonitis with prolonged course despite vigorous treatment using first-line antibiotics, chylous ascites, and elevated ascitic ADA may hint the possibility of mycobacterial infections, and these findings suggest investigations aiming at mycobacterium. However, the diagnosis is usually difficult due to lack of prompt, accurate, and inexpensive approaches. There has been no standard antibiotic protocol, and a combination of multiple appropriate antibiotics is warranted. Timely catheter removal is necessary if antimicrobial therapy is not rewarding. The prognosis can be extremely poor. Efforts should be made to better define the prophylaxis, pathogen identification, and treatment.

## Data Availability

The patient results used and/or analyzed during the current case report are available from the corresponding author on reasonable request.

## Abbreviations

AIDS: Acquired immune deficiency syndrome
ADA: Adenosine deaminase
AFB: Acid-fast bacilli
CT: Computed tomography
ESKD: End stage kidney disease
MAC: *M. avium* complex
*M.*: *Mycobacteria*
NTM: Nontuberculous mycobacteria
PD: Peritoneal dialysis
TG: Triglyceride
WCC: White cell count
